# Supplementary material for: Cytogenetic characterization and mapping of the repetitive DNAs in Cycloramphus bolitoglossus (Werner, 1897): More clues for the chromosome evolution in the genus Cycloramphus (Anura, Cycloramphidae)
Source: PLoS One. 2021 Jan 13;16(1):e0245128. doi: 10.1371/journal.pone.0245128 (PMC7806164; doi:10.1371/journal.pone.0245128)
Supplement: S1 Table — (DOCX) [file pone.0245128.s001.docx]

**S1 Table**. **5S rDNA accession numbers.**

| **5S rDNA sequences** | **GenBank accession numbers** |
| --- | --- |
| *Xenopus laevis* | J01009, J01899, K02695, M10676, M10850, M35055, M35175, M35176 and M30904 |
| *Xenopus laevis oocyte* | J01898, J01012, J01010, M10027, M10635, M63899 and X05089 |
| *Xenopus tropicalis* | NR_033271 and X12622 |
| *Xenopus tropicalis oocyte* | NR_033270, X12623 and X12624 |
| *Xenopus borealis* | K01374, K01537, V01425 and V01426 |
| *Amolops mantzorum* | KX913750, KX913753, KX913756- KX913758, KX913762 and KX913778 |
| *Gatrotheca riobambae* | M74438 |
| *Pelophylax lessonae* | FJ572051 |
| *Pelophylax ridibunda* | FJ572052 |
| *Lithobates pipiens* | X58368 |
| *Lithobates_catesbeianus* | X58367 |
| *Anaxyrus_americanus* | X58365 |
| *Engystomops petersi 5S type I* | JF325859, JF325866 and JF325867 |
| *Engystomops petersi 5S type II* | JF325847 and JF325858 |
| *Engystomops freibergi 5S type I* | JF325868 - JF325870. |
| *Engystomops freibergi 5S type II* | JF325844 and JF325845 |
| *Pseudis tocantins 5S type I* | [KX170899.1](https://www.ncbi.nlm.nih.gov/nucleotide/KX170905.1?report=genbank&log$=nucltop&blast_rank=2&RID=9VN1ETX2014) and [KX170901.1](https://www.ncbi.nlm.nih.gov/nucleotide/KX170905.1?report=genbank&log$=nucltop&blast_rank=2&RID=9VN1ETX2014) |
| *Pseudis tocantins 5S type II* | [KX170905.1](https://www.ncbi.nlm.nih.gov/nucleotide/KX170905.1?report=genbank&log$=nucltop&blast_rank=2&RID=9VN1ETX2014) and [KX170906.1](https://www.ncbi.nlm.nih.gov/nucleotide/KX170905.1?report=genbank&log$=nucltop&blast_rank=2&RID=9VN1ETX2014) |
| *Physalaemus cuvieri 5S type I* | JF281127, JF281128, JF281130 and JF281131 |
| *Physalaemus cuvieri 5S type II* | JF281132 |

Sequences of 5s rDNA from GenBank used in the comparative analysis in the present work.
