# Supplementary material for: Cytogenetic characterization and mapping of the repetitive DNAs in Cycloramphus bolitoglossus (Werner, 1897): More clues for the chromosome evolution in the genus Cycloramphus (Anura, Cycloramphidae)
Source: PLoS One. 2021 Jan 13;16(1):e0245128. doi: 10.1371/journal.pone.0245128 (PMC7806164; doi:10.1371/journal.pone.0245128)
Supplement: S2 Table — (DOCX) [file pone.0245128.s002.docx]

**S2 Table**. **PcP190 satDNA accession numbers.**

| **PcP190 sequences** | **GenBank accession numbers** |
| --- | --- |
| *Physalaemus albifrons* | KM361694.1 - KM361698.1 |
| *Physalaemus albonotatus* | KM361689.1 - KM361693.1 |
| *Physalaemus cuvieri* | JF281109 - JF281125 and KM361673.1 - KM361683.1 |
| *Engystomops freibergi* | MK491531.1 and MK491533.1 - MK491535.1 |
| *Physalaemus centralis* | KM361684.1 - KM361688.1 |
| *Physalaemus ephippifer* | KM361699.1 and KM361700.1 |
| *Physalaemus marmoratus* | KM361701.1 - KM361706.1 |
| *Leptodactylus latrans* | KM361718.1 - KM361724.1 |
| *Crossocadctylus gaudichaudii* | KM361725.1 and KM361726.1 |
| *Pseudis tocantins PcP-1a* | KX170908, KX170909, KX170931, KX170887, KX170895 and KX170896 |
| *Pseudis tocantins PcP-1b* | KX170911 - KX170920 |
| *Pseudis tocantins PcP-2* | KX170921 - KX170930 and KX170897 |
| *Pseudis tocantins PcP-3* | KX170931 - KX170933 |
| *Pseudis tocantins PcP-4* | KX170887 - KX170889 |
| *Pseudis tocantins PcP-5* | KX170890, KX170892 and KX170898 |
| *Pseudis tocantins PcP-6* | KX170891 |
| *Pseudis tocantins PcP-7a* | KX170892 - KX170894 |
| *Pseudis tocantins PcP-7b* | KX170892, KX170895 - KX170898 |

Sequences of PcP190 satDNA from GenBank used in the comparative analysis in the present work.
