# Supplementary material for: Cytogenetic characterization and mapping of the repetitive DNAs in Cycloramphus bolitoglossus (Werner, 1897): More clues for the chromosome evolution in the genus Cycloramphus (Anura, Cycloramphidae)
Source: PLoS One. 2021 Jan 13;16(1):e0245128. doi: 10.1371/journal.pone.0245128 (PMC7806164; doi:10.1371/journal.pone.0245128)
Supplement: S3 Table — (DOCX) [file pone.0245128.s003.docx]

| CHROMOSSOME | Lenght^a^  $\boldsymbol{(}\bar{\boldsymbol{x}}\boldsymbol{\pm\sigma}$) | % Lenght^b^ $\boldsymbol{(}\bar{\boldsymbol{x}}\boldsymbol{\pm\sigma}$) | *q* Length^c^  $\boldsymbol{(}\bar{\boldsymbol{x}}\boldsymbol{\pm\sigma}$) | *p* Lenght^d^  $\boldsymbol{(}\bar{\boldsymbol{x}}\boldsymbol{\pm\sigma}$) | Arm Ratio*^e^* $\boldsymbol{(}\bar{\boldsymbol{x}}\boldsymbol{\pm\sigma}$) | Centromere index^f^  $\boldsymbol{(}\bar{\boldsymbol{x}}\boldsymbol{\pm\sigma}$) | Morphology Abreviation |
| --- | --- | --- | --- | --- | --- | --- | --- |
| 1 | 15.23 ± 3.33 | 15.50 ± 0.70 | 9.10 ± 1.95 | 6.12 ± 1.64 | 1.52 ± 0.25 | 0.400 ± 0.041 | Metacentric |
| 2 | 12.70 ± 3.59 | 12.76 ± 1.36 | 8.72 ± 2.38 | 3.97 ± 1.93 | 2.62 ± 1.48 | 0.309 ± 0.094 | Submetacentric |
| 3 | 10.47 ± 2.23 | 10.71 ± 1.01 | 6.62 ± 1.27 | 3.84 ± 1.24 | 1.84 ± 0.47 | 0.360 ± 0.061 | Submetacentric |
| 4 | 8.67 ± 1.96 | 8.94 ± 1.54 | 5.27 ± 1.47 | 3.39 ± 075 | 1.57 ± 0.37 | 0.397 ± 0.061 | Metacentric |
| 5 | 7.64 ± 1.69 | 7.86 ± 1.34 | 4.44 ± 0.99 | 3.19 ± 1.10 | 1.52 ± 0.64 | 0.413 ± 0.078 | Metacentric |
| 6 | 6.46 ± 1.31 | 6.63 ± 0.67 | 3.6 ± 0.70 | 3.56 ± 1.46 | 1.16 ± 0.31 | 0.483 ± 0.076 | Metacentric |
| 7 | 6.02 ± 1.40 | 6.14 ± 0.54 | 3.46 ± 0.76 | 2.81 ± 0.90 | 1.29 ± 0.28 | 0.443 ± 0.053 | Metacentric |
| 8 | 5.70 ± 1.49 | 5.75 ± 0.45 | 3.45 ± 0.97 | 2.45 ± 0.79 | 1.43 ± 0.33 | 0.417 ± 0.055 | Metacentric |
| 9 | 5.50 ± 1.47 | 5.53 ± 0.46 | 3.17 ± 0.93 | 2.44 ± 0.77 | 1.32 ± 0.28 | 0.436 ± 0.053 | Metacentric |
| 10 | 5.35 ± 1.55 | 5.36 ± 0.59 | 3.03 ± 0.87 | 2.41 ± 0.87 | 1.33 ± 0.44 | 0.440 ± 0.070 | Metacentric |
| 11 | 5.21 ± 1.48 | 5.23 ± 0.56 | 2.99 ± 0.84 | 2.22 ± 0.81 | 1.42 ± 0.49 | 0.424 ± 0.071 | Metacentric |
| 12 | 4.92 ± 1.46 | 4.92 ± 0.55 | 2.76 ± 0.75 | 2.16 ± 0.89 | 1.40 ± 0.54 | 0.432 ± 0.078 | Metacentric |
| 13 | 4.61 ± 1.48 | 4.61 ± 0.72 | 2.71 ± 0.86 | 1.90 ± 0.80 | 1.55 ± 0.67 | 0.411 ± 0.081 | Metacentric |
| Chromosome measurements with average and standard deviation of ten metaphases of *C. bolitoglossus* for karyotype and nomenclature and classification accordingly with the centromeric position on mitotic chromosomes proposed by Green and Sessions (1991).  *a* Total length of chromosome *b* Percent length = 100 x length of chromosome ÷ total length of haploid genome.  *c* Length of long arm  *d* Length of short arm  *e* Length of long arm ÷ length of short arm.  *f* Length of short arm ÷ total chromosome length | | | | | | | |

**S3 Table**. **Chromosomes measurements.**
