# Supplementary material for: Cytogenetic characterization and mapping of the repetitive DNAs in Cycloramphus bolitoglossus (Werner, 1897): More clues for the chromosome evolution in the genus Cycloramphus (Anura, Cycloramphidae)
Source: PLoS One. 2021 Jan 13;16(1):e0245128. doi: 10.1371/journal.pone.0245128 (PMC7806164; doi:10.1371/journal.pone.0245128)
Supplement: S4 Table — (DOCX) [file pone.0245128.s004.docx]

**S4 Table.** **PcP190 SatDNA genetic similarity**

| Species | Similarity (%) |
| --- | --- |
|  | *C. bolitoglossus* PcP |
| *C. bolitoglossus* | 94.86 |
| *Physalaemus ephippifer* | 89.06 |
| *Physalaemus marmoratus* | 89.60 |
| *Physalaemus albonatus* | 89.06 |
| *Pseudis minuta* | 87.49 |
| *Crossocadctylus gaudichaudii* | 87.08 |
| *Pseudis limellum* | 86.87 |
| *Physalaemus centralis* | 86.01 |
| *Leptodactylus latrans* | 85.71 |
| *Physalaemus albifrons* | 85.01 |
| *Physalaemus cuvieri* | 84.59 |
| *Pseudis tocantins* | 82.74 |
| *Pseudis bolbodactyla* | 81.86 |
| *Engystomops freibergi* | 81.50 |
| *Pseudis parva* | 80.86 |
| *Pseudis fusca* | 77.94 |
| CB5STIIb | 59.50 |
| CB5STIIc | 59.10 |
| CB5STIIa | 42.40 |
| CB5SPG | 39.20 |

Genetic similarity (%) between sequences of PcP190 Sat DNA from *C. bolitoglossus* and those from anurans obtained from GenBank database and PcP190 Sat DNA and 5S rDNA from *C. bolitoglossus*.
