# Supplementary material for: Cytogenetic characterization and mapping of the repetitive DNAs in Cycloramphus bolitoglossus (Werner, 1897): More clues for the chromosome evolution in the genus Cycloramphus (Anura, Cycloramphidae)
Source: PLoS One. 2021 Jan 13;16(1):e0245128. doi: 10.1371/journal.pone.0245128 (PMC7806164; doi:10.1371/journal.pone.0245128)
Supplement: Attachment — Submitted filename: Dear authors.docx [file pone.0245128.s005.docx]

Dear authors, I point some doubts:

Page 1, line 9: Standardize authors' addresses. I suggest following their formats of address 3 or 5.

Page 10, line 230: What is the classification of chromosome pair 12?

Page 10, line 233: I suggest organizing the results in figure 1 in the same order as the text. I also suggest that karyotypes with a white background be at the top and black ones at the bottom of the image.

Page 10, line 233: Better explain the repetition of pair 6 in figure 1C.

Page 10, line 234: An arrow is missing in the heterochromatic region of chromosomal pair 4 in figure 1B.

Page 10, line 237: Did not observe the interstitial heterochromatic marking on chromosome pair 8 and 10? How many metaphases were analyzed to identify this interstitial mark?

Page 10, line 238: The metaphases of figure 1D, 1E and 1F were also stained with DAPI? Why didn't chromosome pair 6 show negative DAPI?

Page 10, line 239: The results of the probes in the text do not follow the order of metaphases in the image! Check.

Page 10, line 240: How to differentiate subterminal markings from terminals?

Page 10, line 241: Check the centromeric markings in pairs 2, 7, 8, 9, 10 and 12 in figure 1E! Did pairs 3, 4, 5, 6 and 13 not show markings? what is the marking pattern of par 11? Did pair 9 show two markings? Explain the marking standards better.

Page 10, line 224: I suggest cleaning figure 1F better. Check the pericentromeric and centromeric term used in the text. It was previously indicated that heterochromatin is present in the pericentrometic region. In this sense, did the (GACA)_4_ probe hybridize in both the heterochromatin and euchromatin regions? Because pair 1 does not have heterochromatin in the terminal region of the larger chromosomal arm.

Page 11, line 246: check the results of figure 1D. The figure shows results of the telomeric probe and the probe text (GATA) 8.

Page 11, line 247: “add (GAA)_10_”.

Page 11, line 252: I suggest following an order in the presentation of the figures. We move from figure 1 to 3!

Page 13, line 282: I didn't find figure 2C and D.

Page 13, line 288: Explain figure 4c and 4d in the text.

Page 14, line 300: I didn't find tables S1 and S2 in the text.

Page 28, line 714: In figure 6 what do the gray and red circles mean? What is the difference between red circles with lines and without lines?
