# Supplementary material for: Cytogenetic characterization and mapping of the repetitive DNAs in Cycloramphus bolitoglossus (Werner, 1897): More clues for the chromosome evolution in the genus Cycloramphus (Anura, Cycloramphidae)
Source: PLoS One. 2021 Jan 13;16(1):e0245128. doi: 10.1371/journal.pone.0245128 (PMC7806164; doi:10.1371/journal.pone.0245128)
Supplement: Attachment — Submitted filename: Response_to_Reviewers.docx [file pone.0245128.s006.docx]

Author response

Our responses to the suggestions of the reviewers are presented below. We would like to thank all the reviewers for their important contributions for the improvement of the manuscript. The corrections are highlighted in the text of the manuscript.

Cordially,

Daniel Pacheco Bruschi

**Reviewer #1:**

*The authors performed some molecular cytogenetic analysis in the frog Cycloramphus bolitoglossus. The main goal of the work “to understand the chromosome shifts that have occurred during the evolutionary history of this group” is unfortunately not fully achieved. The main goal of this paper is to describe the karyotype and repetitive DNA arrangement on Cycloramphus bolitoglossus providing data for future studies. We also contributed with this comprehension based on data already available about this genus (de Sá et al. 2020, Noleto et al. xxxxx), but we know that more data and analysis are still necessary to uncover the major chromosome changes along the evolution of the genus Cycloramphus. However, our study is the first to show the chromosome changes in Cycloramphus anchored in the most recent phylogenetic hypothesis for the genus. This approach helped to understand some pleisiomorphic conditions that will be useful in future studies involving phylogenetic, evolutionary and taxonomic approaches.The authors analyze just one species and made several phylogenetic inferences about possible evolutionary pathways inside the genus, but as all closely related species lack similar cytogenetic analysis (as stated by the authors lines 82-84), the conclusions are too speculative.*

**R:** Despite of the fact that we sampled just one species, we used several cytogenetic data already available for the genus *Cycloramphus*, as well as for *Zachaenus* and *Thoropa* (species closely related to *Cycloramphus*). Additionally, we based our inferences in the light of the phylogenetic relationships of the genus provided by de Sá et al. (2020). In fact, there is a lack of cytogenetic data for several species of *Cycloramphus*, but from the five species, of the clade that include *C. bolitoglossus,* only two species lack cytogenetic information. In this case, the use of a most parsimonious interpretation on the data (ours and those already available in the literature) can be used to understand the changes on pair 1 of *C. fuliginosus*. Since all other *Cycloramphus* species already studied cytogenetically have a metacentric pair 1, the most parsimonious interpretation is that this is the pleisiomorphic condition for the genus and the change occurred at least in the *C. fuliginosus* linage, since its sister taxa (*C. brasiliensis*) have a metacentric pair 1. Additionally, almost all of the species of *Cycloramphus* studied cytogenetically possess the NORs, or the secondary constrictions, in the pair 6. However, *C. acangatan* has the NOR sites in the chromosome 1. The most parsimonious way to solve this transformation is that the pleisiomorphic condition for NOR sites in *Cycloramphus* is their location in pair 6.

*“The use of novel chromosomal markers provided new perspectives for the investigation of chromosomal evolution in the genus Cycloramphus”: In fact, they CAN add a new chapter into this story IF also applied to more species. Why haven´t the authors perform such analysis in more species from this genus?*

**R:** Suggestion accepted. About the novel chromosome markers, we agree with the reviewer and we changed the text of the pointed section by the reviewer. Additional information would be the best scenario. However, several species of *Cycloramphus*, such as *C. stejnegeri*, *C. faustoi* and *C. bolitoglossus*, are under threat (see IUCN list). Because of this, collection of individuals of *Cycloramphus* needs careful attention from both, researches and governmental agencies that issues authorizations for this. However, despite the fact that we sampled just one species, there are sufficient cytogenetic data available in the literature to perform comparative analysis.

*Besides, Title is very descriptive, since it must contain the real findings of this papers. In the same way abstract is also very descriptive without a clear central line of objective/conclusions.*

**R:** Suggestion accepted. Title and abstract were reviewed and we rewrote them, and conclusions are now highlighted.

*Some figures are also strange/incomplete and must be arranged: eg. Fig1D and Fig4A the pair are not properly aligned;*

**R:** Suggestion accepted. We upload new figure design.

*Fig 1C, why there is a bigger chromosomal pair named as “6” in the end of the karyotype?*

**R:** Suggestion accepted. We improved the layout of the figure to solve this question. Because the pair 6 of our best metaphase was in a low quality and is a NOR-bearing chromosome, we realized that emphasis on this pair with a higher quality image, will allow readers to see it better.

Fig1F is probably both incomplete and of terrible quality and must be replaced.

**R:** Suggestion accepted. We replaced the 1F figure.

**Reviewer #2**

***Abstract.***

*Line 51- ...’the pattern of DNA repetitive classes’… In my opinion, it should be added the word ‘some’ before DNA, as in this study are analyzed only two repetitive DNA classes plus seven microsatellites.*

**R:** Suggestion accepted. We added this information in the sentence.

*Line 55- Please provide the FN of C. bolitoglossus in the abstract.*

**R:** FN of *C. bolitoglossus* was added.

*Lines 64-67- The authors should add a brief description here of PcP190 occurrence in other groups of Anura, or at least state that this sequence generally shows a hypervariable region and a more conserved one*.

**R:** Suggestion accepted. We added the information of PcP190 occurrence among anurans.

*Line 67- ”our data showed/have shown evidence of”? Please check this sentence.*

**R:** Sentence was reviewed.

***Keywords***

*The word repetitive DNA is on the title and may be replaced, although keywords are not included in the final version of the manuscript.*

**R:** Suggestion accepted. We replaced this sentence for “satellite”.

***Introduction.***

*Line 82- I think that it is more appropriate to state the number of studied species instead of ‘Few’.*
**R**: Suggestion accepted. Sentence was reviewed and we added the number of species studied cytogenetically so far.

*Line 84- The diploid number (2n=26) is observed in all species. The word “almost” should be removed or the sentence modified, as some species are showing non bi-armed chromosomes.
As a suggestion: “The diploid number (2n=26) is conserved/observed in all the species, with karyotypes that are generally composed of metacentric and submetracentic pairs”.*

**R**: Suggestion accepted. Sentence was reviewed and we followed the reviewer suggestion.

*Line 85- the word submetacentric is misspelled*.

**R:** Suggestion accepted. We corrected the word in the reviewed version.

*Lines 87-88- To date, there were studied ten species of Cycloramphus (Noleto et al., 2011). Three of them showed FN=50 while the others a FN=52. Other species with FN=52 should be also included in this statement as there were named only two of them.*

**R:** Suggestion accepted. Sentence was reviewed and we followed the reviewer suggestions.


*Lines 95-101. Why including 45S in this paragraph? Although being rDNA this sequence was not studied here and should be removed.*

**R:** Suggestion accepted. We removed the mention on 45S rDNA in the reviewed version of the manuscript.

***Material and Methods***

*Line 139- Specimens were deliberately collected, independently of their low abundance. The word “accidentally” should be removed.*

**R:** Suggestion accepted. We removed this word from the manuscript. We also provided more information about the collection procedures.

*Line 154- Howell is misspelled*

**R:** Suggestion accepted. We corrected in the new version of the manuscript.

*Line 155- Sumner is misspelled*

**R:** Suggestion accepted. We corrected in the new version of the manuscript.

*Line 166- The PcP190 sequence was also amplified with this protocol; it should be included here or describe the PCR protocol used for PcP190. Moreover, if possible, provide the annealing temperatures used for both sequences.*

**R:** Suggestion accepted. We corrected this issue in the new version of the manuscript.

*Line 167- “110x PCR Buffer”. Please correct this, and the word buffer should be without a capital letter.*

**R:** Suggestion accepted. We corrected the buffer concentration in the revised version.

*Lines 173-174- Please provide the manufacturers of these kits.*

**R:** Suggestion accepted. Manufacturers information was added in the new version.

*Lines 177 and 178- The words forward and reverse, including colons, should be without italics.*

**R:** Suggestion accepted. This section was rewritten in the new version.

*Lines 188-189- I think that comparisons between these four groups of sequences are not exclusively restricted to C. bolitoglossus, are they? If not, please state if in a different manner.*

**R:** Suggestion accepted. Some of the comparisons were made only between sequences from *C. bolitoglossus*, while other comparisons used *C. bolitoglossus* and sequences available on GenBank. We reformulate this section pointing this.

*Lines 202, 209- ”(Roche)”. In order to follow the same criterion, please provide the country information as before.*

**R:** Suggestion accepted. We corrected this issue and followed the reviewer suggestions for all the manufacturers in the text.

*Line 2013- same comment for Sigma-Aldrich.*

**R:** Suggestion accepted.

*Line 220- “…(0.4μg/Ml)”, please correct “Ml”.*

**R:** Suggestion accepted. Unit measure is corrected in the new version.

***Results.***

*Lines 229-231. At this point, a question arises. How did you measure chromosomes in order to determine their chromosome morphology and what criterion did you adopt? I recommend measuring them using one of the non-commercial software Drawid or Micromeasure, or other, and use the nomenclature proposed by Green and Session (1991) for anuran chromosomes. Without measurements, chromosome morphology becomes very subjective and can affect phylogenetic interpretations. For example, in my opinion, chromosomes of pair 1 are “more metacentric” than those chromosomes of pair 5. However, you considered them as submetacentric and metacentric, respectively.
Moreover, in the Discussion section (Lines 329-331), it is discussed about a metacentric morphology for pair 1 would be plesiomorphic for Cycloramphus. Although results and interpretations would possibly remain unchanged, this is important for using the same criteria among cytogeneticists, and sometimes also provide interesting subtle differences when comparing species karyotypes.*

**R:** Suggestion accepted. Thanks for these suggestions; indeed, we had not performed chromosome measurements before. We followed yours suggestion and in the revised version of the manuscript we added chromosome measures (a supplementary table [sup. table 3] was added to show this data). Because of this we realized that pair 1 of *C. bolitoglossus* is actually metacentric, as pointed by the reviewer. We corrected this in results and we reformulated the discussion in the section pointed by the reviewer.

*Line 233- Figure 1C is referenced before Figure 1B (line 237). This must be corrected.*

**R**: Suggestion accepted. We corrected this issue in the new version of the manuscript.

*Lines 233-238- The authors applied DAPI after C-bands in order to enhance resolution. In my experience, this works, although this method allows detecting bands that sometimes are undetectable by the conventional C-band protocol, for example, the conspicuous heterochromatic bands observed in the pericentromeric region of pair 1.
As a suggestion, to avoid future inconsistencies in the literature or errors in the interpretation of chromosomal characters in other studied species Cycloramphus, authors should emphasize in this paragraph that these results correspond to C-bands+DAPI (i.e. DAPI positive marks after C-bands). Moreover, the first and only mention in this paragraph of the fluorochrome DAPI is between Lines 237-238, stating that “Negative DAPI staining was also observed in pair 6…”.*

**R:** Suggestion accepted. We followed the reviewer suggestions and we added the information regarding C-band+DAPI procedure.

*Lines 245-246- “Chromosome 1 also presented hybridization signals of the (GATA) 8 probe in the terminal region of the long arm (Figure 1D).”
However, (GATA)8 FISH is not shown, and the Figure 1D is the FISH with the telomeric probe. I am right?*

**R:** Suggestion accepted. We corrected this issue in the main text.

*Lines 248-249- “The telomeric probe hybridized all the telomeres found in the chromosomes (Figure 1D).”
Please check Figure 1 citing order. Regardless of whether Figure 1 has been cited as a whole, Figure 1D must be quoted before in the text or change it reference order. The same to Figure 1B.*

**R:** We corrected this in the new version of the manuscript.

*Line 250- I can't visualize some of the ITSs bands described or distinguish them from other non-ITS marks. Is there any possibility to increase the quality of this figure or use insets of the described ITS bands? For instance, the pericentromeric ITS described for homologs of pair 10. Is it possible that the left chromosome of this pair is inverted?*

**R:** Suggestion accepted. We reformulate the Figure 1D by adding arrows indicating the ITSs in the chromosomes. We also corrected the pair 10 in this figure, as suggested by the reviewer.

*Line 252- From which specimen/s did the 5S and PcP190 sequences come from? All specimens where molecularly studied? I can not find in the text, and I think is relevant or at least knowing the sex of the specimens from which the sequences come.*

**R:** Suggestion accepted. We agree that this is necessary; we pointed in the main text the sex and which individuals we used to isolate the sequences of 5S rDNA and PcP190 satDNA. This information is now present in Material and Methods section about the isolation of repetitive DNA sequences.

*Table 1. Why not sorting this table in ascending / descending order of similarity (%)?*

**R:** Suggestion accepted. We reformulated in the main text the Table 1 in a descending order of similarity.

*Lines 287-288- “The CB5STIIa.C1 probe detected a signal in the centromeric region of the homologs of pairs 1 and 4 (Figure 4B),…”
Again, when detecting variation, in my opinion, it is important to clarify in which sample or at least indicate in the legend of the figure in which sex the experiments were carried out. This has the goal that this information is explicitly established for future studies.*

**R:** Suggestion accepted. We reformulated the main text in and we specified the sex of the individuals used in FISH experiments.

*S3 Table Remarks. Should be PcP190 or PcP-Cbol instead of PcP.*

**R:** Suggestion accepted. We included the information “PcP-Cbol” in the actual S4 Table (S3 table in the previous version).

**Discussion**

*Lines 329-331- How was adjusted the morphology of chromosomes of pair 1 or what criterion did you follow? Again, as I recommended in the Results section, without measuring chromosomes, it is hard to speculate. Why not considering pair 1 of C. bolitoglossus as metacentric at first and avoid this speculation? In my opinion, the most conspicuous difference regarding pair 1 is that one observed in C. fuliginosus by Noleto et al., which seems to be an autapomorphic trait. Moreover, as you stated, the metacentric morphology is apparently homeologous among species and plesiomorphic.*

**R:** Suggestion accepted. In the new version of the manuscript, we added a S3 Table showing chromosome measurements and morphology classification of the chromosomes. We also reformulated the inferences regarding the changes in pair 1 of *Cycloramphus* in the main text of the discussion, as pointed by the reviewer.

*Lines 335-338- I disagree in part with this statement. I cannot perceive a difference between chromosomes of pair 1 shown in this study for C. bolitoglossus than those present in C. acangatan and C. eleutherodactylus for example, which are ‘metacentric’.*

R: Suggestion accepted. We agree with the reviewer and we reformulated our inferences about the pair 1 evolution based on chromosome measurement data suggested by the same reviewer.

*Line 380- At this point I have a question regarding the nomenclature of Sat DNAs. First, in other species it is often used a nomenclature that denotes Species_MonomerLength (e.g., PcP190). Second, the sequence found in this species shows > 80% similarity with those found in other anurans, which possibly corresponds to a new variant of PcP190. Why naming this new variant as PcP-Cbol-1 instead of, for example, PcP190-Cbol-1?*

**R:** Suggestion accepted. We have not thought before in change the name of the satellite DNA as a manner to refer to the original work that isolated this sat DNA family first. However, we agree that it is necessary to name the sat DNA to refer the species were it was isolated. Because of this, we changed the name for PcP-Cbol-1 as suggested by the reviewer.

*Lines 339-402- Interesting. PcP190 usually has a centromeric position and, if I am not wrong, this is the first time that this sequence maps on a non-centromeric region of a ‘non-sex’ chromosome. Maybe, this should be highlighted.*

**R:** Suggestion accepted. Indeed, it is an interesting finding that PcP190 mapped in several non-heterochromatic regions. We highlighted this fact in the paragraph of line 429, as suggested by the reviewer.

*Fig 1. B. Should say: C-Bands + DAPI*

**R:** Suggestion accepted. Information of C-bands+DAPI was added to the actual Figure 1C (former Fig. 1B).

**Reviewer #3**

*Page 1, line 9: Standardize authors' addresses. I suggest following their formats of address 3 or 5.*

**R:** Suggestion accepted. Authors affiliation were standardized in the new version of the manuscript as suggested.

*Page 10, line 230: What is the classification of chromosome pair 12?*

**R:** Suggestion accepted. Pair 12 is metacentric, we added this information in the main text (Results section) and in the new version we provide a supplementary table with chromosome measurements and morphology classification of *C. bolitoglossus* chromosomes.

*Page 10, line 233: I suggest organizing the results in figure 1 in the same order as the text. I also suggest that karyotypes with a white background be at the top and black ones at the bottom of the image.*

**R:** Suggestion accepted. We followed the reviewer suggestions and the Figure 1 was reformulated in the new version of the manuscript.

*Page 10, line 233: Better explain the repetition of pair 6 in figure 1C.*

**R:** Suggestion accepted. We reformulated the Figure 1. Actual Figure 1B (former Figure 1C) shows an inset of a better quality chromosome pair 6 to show better the NOR sites.

*Page 10, line 237: Did not observe the interstitial heterochromatic marking on chromosome pair 8 and 10? How many metaphases were analyzed to identify this interstitial mark?*

**R:** Suggestion accepted. Actually, we pointed with arrows those regions in the Figure 1B (actual Figure 1C). We analyzed about 10 metaphases that allowed the identification of this heterochromatic regions. However, we added insets for pair 8 and 10 from other metaphases that are with a better quality.

*Page 10, line 238: The metaphases of figure 1D, 1E and 1F were also stained with DAPI? Why didn't chromosome pair 6 show negative DAPI?*

**R:** Suggestion accepted. Figures 1D, 1E and 1F are from FISH procedure. Former Figure 1B (actual figure 1C) shows the results from C-band followed to DAPI staining procedure. BaOH treatment of C-banding protocol We did not made explicit the information of that chromosome preparation for former Figure 1B result from the C-banding protocol before DAPI staining. In the new version of the manuscript we made this explicit.

*Page 10, line 239: The results of the probes in the text do not follow the order of metaphases in the image! Check.*

**R:** Suggestion accepted. We reformulated both, figures and the main text in the new version of the manuscript.

Page 10, line 240: How to differentiate subterminal markings from terminals?

**R:** Actually, terminal marks is the only visible region of the chromosome in the power resolution of this microscopy. We interpret this question as a recommendation and accepted the suggestion. We rewrote the text.

*Page 10, line 241: Check the centromeric markings in pairs 2, 7, 8, 9, 10 and 12 in figure 1E! Did pairs 3, 4, 5, 6 and 13 not show markings? what is the marking pattern of par 11? Did pair 9 show two markings? Explain the marking standards better.*

**R:** Suggestion accepted. We followed the reviewer suggestions and we added a more detailed description of this results in the new version of the manuscript.

*Page 10, line 224: I suggest cleaning figure 1F better. Check the pericentromeric and centromeric term used in the text. It was previously indicated that heterochromatin is present in the pericentrometic region. In this sense, did the (GACA)_4_ probe hybridize in both the heterochromatin and euchromatin regions? Because pair 1 does not have heterochromatin in the terminal region of the larger chromosomal arm.*

**R:** Suggestion accepted. We followed reviewer suggestions and a better quality image is presented in the Figure 1F in the new version of the manuscript. We also made some changes in the main text indicating that not only heterochromatic regions hybridized with (GACA)_4_ probe.

*Page 11, line 246: check the results of figure 1D. The figure shows results of the telomeric probe and the probe text (GATA) 8.*

**R:** Suggestion accepted. We reformulate the text and in the new version of the manuscript the results is referred to the right figure.

*Page 11, line 247: “add (GAA)_10_”.*

**R:** Suggestion accepted. . (GAA)_10_ was added in the main text.

*Page 11, line 252: I suggest following an order in the presentation of the figures. We move from figure 1 to 3!*

**R:** Suggestion accepted. We moved the presentation of figures in this section for the new version of the manuscript.

*Page 13, line 282: I didn't find figure 2C and D.*

**R**: Suggestion accepted. We changed this and the figure is now provided. We also changed the Figure number as suggested before.

*Page 13, line 288: Explain figure 4c and 4d in the text*.

**R:** Suggestion accepted.

*Page 14, line 300: I didn't find tables S1 and S2 in the text*.

**R:** Suggestion accepted. Tables S1 and S2 are indicated in the lines 192-193.

*Page 28, line 714: In figure 6 what do the gray and red circles mean? What is the difference between red circles with lines and without lines?*

**R:** Suggestion accepted. In the revised version of the manuscript, we added this information in the Figure legend. Briefly, red circles indicate the NORs sites, black lines indicate heterochromatic regions, and gray circles indicate the presence of secondary constrictions.

**# Editor recommendation**

**R:** Suggestion accepted. The Maximum Likelihood tree was estimating using RAxML (Figure 3).
