# Supplementary material for: Cytogenetic characterization and mapping of the repetitive DNAs in Cycloramphus bolitoglossus (Werner, 1897): More clues for the chromosome evolution in the genus Cycloramphus (Anura, Cycloramphidae)
Source: PLoS One. 2021 Jan 13;16(1):e0245128. doi: 10.1371/journal.pone.0245128 (PMC7806164; doi:10.1371/journal.pone.0245128)
Supplement: Attachment — Submitted filename: Additional Editor Comments.docx [file pone.0245128.s007.docx]

Author response

We attended to all suggestions of the editor and reviewers. We thank all of you for the contributions for the improvement of the manuscript. The corrections are highlighted in the text of the manuscript with track changes.

Cordially,

Daniel Pacheco Bruschi

**Editor Comments**

 *Include authority and year in the taxa the first time it appears in the text.*
**R:** Suggestion accepted. We corrected in the new version of the manuscript.

*Page 6, lines 132-133: “The chromosomes were C-banded, following Sumner [40], to define the heterochromatic pattern. This sentence is awkward, please improve it.*

**R:** Sentence was reviewed.

*Page 15, line 337: What does it mean? Ssp is species or specimens?*

**R:** It means species. We corrected in the new version of the manuscript.

*Page 16, line 356: I did not find in the figure (d and d’).*

**R:** We corrected in the new version of the figure in the manuscript.

*Page 17, lines 383-385: “It is interesting to note, however, except C. acangatan and C. fuliginosus, all the Cycloramphus species that have been karyotyped to date…”. This sentence is awkward, please improve it.*

**R:** Sentence was reviewed.

*Page 19, line 452: Why is “magnus” in quotation marks? I recommend that you check this information, and if necessary use “cf.” or “aff.” in the taxonomic context.*

**R:** Suggestion accepted. We corrected in the new version of the manuscript.

*Page 21, line 480: Please remove “and even”.*

**R:** Suggestion accepted. We corrected in the new version of the manuscript.

*S3 Table: please check English in the legend of table S3*
 **R:** Sentence was reviewed.

**Reviwer #3**

*Lines 61-62: “C.boraceiensis, C.dubius”… add a space after “C.”.*

**R:** We corrected in the new version of the manuscript.

*Line 69: This line break space should be removed.*

**R:** We corrected in the new version of the manuscript.

*Lines 136-137: Drawid … [41] and Green and Session [42] references do not match the numbers in the Reference section. I think that this could be because Drawid’s reference is missing in the Reference section. Please, I strongly recommend rechecking all references in the text but also in the Reference section.*

**R:** We corrected this . We also checked all references in the new version of the manuscript.

*Line 236: “…4 and 5.The…”. Please add a space before “The”*

**R:** We corrected in the new version of the manuscript.

*Line 337: As a suggestion, replace “spp.” with “species”*

**R:** We corrected in the new version of the manuscript.

*Line 379: “…C. bandeirensisThe …”. Please correct this issue.*

**R:** We corrected in the new version of the manuscript.

*Line 392: “sequences (ITSs) in of this pair..”. Please correct this sentence.*

**R:** We corrected in the new version of the manuscript.

*Line 433: “C. bolitoglossus chromosomes,a”. Please correct this.*

**R:** We corrected in the new version of the manuscript.

*Line 463:”…67].The …”. Please correct this.*

**R:** We corrected in the new version of the manuscript.

*Line 466: “…[68]. . Despite its…”. Please correct this.*

**R:** We corrected in the new version of the manuscript.

*Line 626: This line break space should be removed*

**R:** We corrected in the new version of the manuscript.

*Line 677: Please replace reference number “584” with “58”.*

**R:** We corrected in the new version of the manuscript.
